# The value of morphology: osteoclast-like cells in soft tissue tumours

**DOI:** 10.3389/pore.2025.1612175

**Published:** 2025-09-12

**Authors:** Ali Al Khader, Christian Seghetti, Fatine Oumlil, Anna Tollit, Roberto Tirabosco, Fernanda Amary, Paul O’Donnell, Adrienne M. Flanagan

**Affiliations:** ^1^ Department of Microbiology, Pathology, and Forensic Medicine, Faculty of Medicine, The Hashemite University, Zarqa, Jordan; ^2^ Cellular and Molecular Pathology, Royal National Orthopaedic Hospital NHS Trust, Middlesex, United Kingdom; ^3^ Research Department of Pathology, University College London Cancer Institute, London, United Kingdom; ^4^ Department of Experimental Medicine, TOR, University of Rome Tor Vergata, Rome, Italy; ^5^ Department of Radiology, Royal National Orthopaedic Hospital NHS Trust, Middlesex, United Kingdom

**Keywords:** digital pathology, osteoclast, artificial intelligence, mineralisation, soft tissue tumour

## Abstract

Recognition of unusual histological features can augment and hasten a diagnosis but also stimulate ideas about physiological and pathological cellular interactions. Osteoclasts resorb mineralised tissue and therefore can be found at sites of heterotopic bone formation. However, multinucleated giant cells with morphological features of osteoclasts, so called ‘osteoclast-like cells’ can also be encountered in a variety of soft tissue tumours unrelated to ossification and calcification. Prompted by the presence of osteoclast-like cells in undifferentiated pleomorphic sarcoma while undertaking our Artificial Intelligence project for classifying sarcoma, we reviewed the English literature for these cells in soft tissue tumours and we found that this was poorly documented, and much was published before the release of the WHO essential diagnostic criteria in 2020. There were numerous single case reports and small series of a broad range of soft tissue tumours with osteoclast-like cells but only a limited number of diagnoses in which these cells were reported recurrently. We provide a comprehensive update of osteoclast-like cells and mineralisation in soft tissue tumours from the literature. We also present real-world incidence of osteoclast-like cells from selected tumour types in our Whole Slide Image (WSI) library of soft tissue tumours. Assessment of WSI from 1100 different patients showed that osteoclast-like cells were relatively common and under-recognised in nodular fasciitis (18.5 of 200), angiomatoid fibrous histiocytoma (17.5% of 40), undifferentiated pleomorphic sarcoma (15% of 261) and epithelioid sarcoma (9% of 68) while they were never encountered in myxofibrosarcoma (0/250) and clear cell sarcoma of soft tissue (0/80). Awareness of this phenomenon not only helps shape the differential diagnosis but also can be used to stimulate pathobiological questions and to enhance the performance of AI models for classifying disease.

## Introduction

The histopathology field has been revolutionised by the major advances in the understanding of the molecular mechanisms of neoplastic disease, and this is reflected in the changes introduced by the recent World Health Organisation classification of tumours series of Blue Books. In the era of complex molecular and genetic analysis, morphology can be easily overlooked. This can only be to the disadvantage of a great part of the world where access to advanced ancillary testing is, at best, limited, and where morphology and restricted use of immunohistochemistry remains the cornerstone in surgical pathology.

While working on our Artificial Intelligence (AI) project for classifying sarcoma (AI-SCOPE: Artificial Intelligence for SarComa Outcome PrEdiction [1]), we noted that the incidence of osteoclast-like cell was relatively common in several tumours. We identified numerous case reports, but an overview of the subject was not available. This prompted us to review how often osteoclast-like cells and mineralisation (ossification and calcification) were described in soft tissue tumours in the recent WHO classification of tumours series. We also reviewed digitised whole slide images (WSI) of specific diagnoses as part of AI-SCOPE to assess the incidence of these features in soft tissue tumours employing standards of classification based on current WHO Books.

Osteoclasts are haematopoietic cells originating from myeloid progenitors under the stimulation of key factors such as CSF-1 [2] and receptor activator of nuclear factor-kB ligand (RANKL) [3–5]. Their primary function is to maintain a healthy skeleton through bone reabsorption brought about by a tightly regulated physiological process involving interplay between several molecules, ensuring appropriate recruitment to sites requiring bone replacement. Disruption of these processes manifests most commonly as unregulated osteolysis of the skeleton and is seen in common diseases such as osteomyelitis, osteoporosis, and metastatic neoplasms to bone [6] and rarely in primary bone tumours [7, 8] and less commonly in reduced osteoclast numbers and or function, as in osteopetrosis [9].

Osteoclasts in extra-skeletal sites are an abnormal finding, they occur commonly in combination with soft tissue calcification and/or ectopic bone formation, which comes about by the reactivation of bone-forming programmes involving the recruitment of local stem cells to form bone and cartilage, remodelling of the bone through osteoclast activity and the formation of mature bone [10].

**FIGURE 1:**
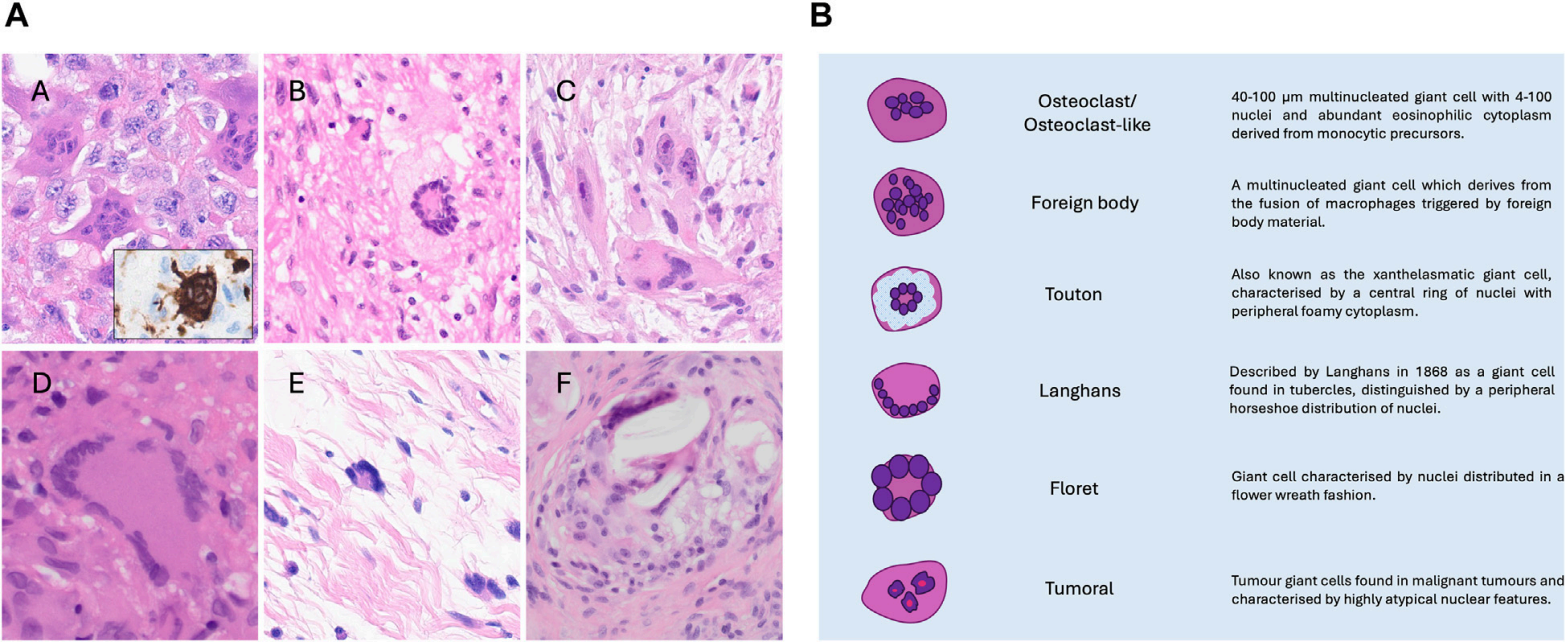
**(A)** Photomicrographs of hematoxylin and eosin-stained sections showing different kinds of multinucleated giant cells. Osteoclast-like giant cells in undifferentiated pleomorphic sarcoma. CD68 immunostain highlights the dendritic processes typical of those cells (A); A touton giant cell in myxofibrosarcoma (B); tumour multinucleate giant cells in myxofibrosarcoma exhibiting atypical pleomorphic nuclei (C); Langhans-type giant cells in a sarcoidosis granuloma (D); floret multinucleated cell in a pleomorphic lipoma (E); foreign body giant cells (F). Cases from the AI Scope library. **(B)** Schematic representation of giant cells.

Heterotopic bone is commonly caused by trauma and ischaemia, resulting in dystrophic calcification, which can be the forerunner of ossification. This process is not uncommonly seen in tumours, but it is a non-specific finding and can happen in virtually all neoplasms. In contrast, mineralisation is an essential criterion for some neoplasms including extra-skeletal osteosarcoma, and calcifying aponeurotic fibroma, amongst others [11, 12]. Autoimmune disease can also account for ectopic mineralisation, exemplified by calcinosis cutis [6, 12, 13]. It has long been recognised that a wide variety of tumours, including many types of carcinoma particularly pancreas, breast and others [14, 15], and also uterine smooth muscle tumours [16], harbour osteoclast-like giant cells generally in the absence of mineralisation. The term osteoclast-like cell is preferred to ‘osteoclast’ as it can be challenging and sometimes impossible to distinguish osteoclasts from the other types of multinucleate giant cells because of their overlapping morphological features; these include so-called foreign body, Tuton and Langhans giant cells (Figure 1). However, osteoclasts uniquely have the ability to resorb bone [17] and historically, this function was employed to define osteoclasts in an experimental setting [17] and was used to establish that the multinucleate giant cells in tenosynovial giant cell tumours (previously known as pigmented villonodular synovitis [18]), pilar tumour of the scalp [19] and giant cell tumour of tendon sheath [20] and in an undifferentiated pleomorphic sarcoma, previously known as malignant fibrous histiocytoma [21], were accurately classified as osteoclasts [22]. Previous studies, summarised in an overview [23] have shown that osteoclasts can also be distinguished from other multinucleate giant cells by the expression of the calcitonin receptor, the vitronectin receptor and tartrate-resistant acid phosphatase and the absence of HLA-DR. However, it is no longer considered valuable to undertake such experimental and immunohistochemistry profiling in a diagnostic setting. From a practical diagnostic perspective, the classification of an ‘osteoclast-rich tumour’ is based on the presence of a large number of such cells and, therefore is not considered a major challenge in distinguishing osteoclasts from other multinucleate cells.

The mechanism explaining the presence of osteoclast-like cells in tumours in the absence of mineralisation is generally unknown. Although RANKL is an important molecule in osteoclast recruitment [24] there is no strong evidence that its expression, in concentration or duration, is the cause of the osteoclast-like cells in these tumours. However, a fusion gene involving CSF-1, resulting in its elevated expression, is found in tenosynovial giant cell tumours explains the presence of osteoclasts in these tumours [25]. More recently, giant cell tumours of soft tissue have been found to harbour a *HMGA2::NCOR2* fusion gene, although it is unknown how this mediates osteoclast-like cell recruitment [26, 27].

Here, using the presence of osteoclast-like cells and mineralisation in soft tissue tumours as an exemplar, we highlight the value of detailing morphological features in tumour pathology. Such information would benefit the majority of pathologists around the world who have at best limited access to advanced technologies, including immunohistochemistry and next-generation sequencing, in delivering their clinical practice. In the context of AI, which is rapidly being introduced into surgical pathology, morphology is far from obsolete because AI algorithms for disease classification are mainly trained on haematoxylin and eosin-stained digitalised slides. Although large pathology foundation models are self-supervising, thereby overcoming the need for annotation of images by pathologists [28–30], training models for classifying rare diseases, such as sarcoma and its mimics, will likely require annotation by pathologists for some time to come [31]. This underscores the importance of the identification of morphological features as described in the manuscript. Lastly, appreciation of histological features both stimulates and allows the analysis of mechanisms of disease to be investigated.

## Materials and methods

### Literature revision

A list of soft tissue tumours (Supplementary Table S1) was obtained from the latest editions of the WHO Classification of Tumours series of books including Soft tissue and bone tumours (2020), Digestive system tumours (2019), Skin tumours (beta online version) Breast tumours (2019), Head and neck tumours (2023), Eye and orbit tumours (beta online version), Thoracic tumours (2021), Urinary and male genital tumours (2024), Paediatric tumours (2022) and Haematolymphoid tumours (2024) [32–41]. In addition, we included three emerging entities [42–44] not found in the WHO books, which are documented as containing osteoclasts of which we are aware from our diagnostic practice, published literature and data accrued from the North Thames Genome Laboratory Hub. Mesenchymal tumours originating from bone and uterus were not included.

The presence of “osteoclast-like cells” and mineralisation, including ossification and calcification, was sought in the histopathology description in the WHO books listed above and recorded (Supplementary Table S1). For each listed diagnosis, we interrogated the English literature using PubMed and Google Scholar for records of osteoclast-like cells and mineralisation in soft tissue tumours.

### Case review and data collection

We reviewed Whole Slide Images (WSI) from cases from England and Wales included in our AI SCOPE library (one or two slides per case) built with tumours classified according to the essential criteria in the current WHO Books along with at least one confirmatory ancillary testing, such as immunohistochemistry, fluorescence *in situ* hybridisation (FISH), or next-generation sequencing (NGS), for example, detection of a *USP6* rearrangement in nodular fasciitis. Additional diagnoses were also reviewed for reasons described in the Results section.

Recognising the challenge in distinguishing osteoclasts from different forms of multinucleate cells, we have employed the term ‘osteoclast-like’ giant cells in the tumours studied. To mitigate further misclassification, clusters of such cells were required to be identified within a tumour. Figure 1 shows the different forms of multinucleate cells and describes their distinguishing features.

Ethical approval was given for undertaking the study “An Artificial Intelligence (AI) solution for diagnosing, prognosticating as well as predicting outcome of sarcomas and their mimics: a multi-centre study.” IRAS project ID: 328987 Protocol number: EDGE 161548. REC reference: 23/NI/0166. Sponsor University College London has been approved by HRA and Health and Care Research Wales (HCRW) (14th December 2023) and by Health and Social Care Research Ethics Committee B (HSC REC B) Office for Research Ethics Committees Northern Ireland (ORECNI) Lissue Industrial Estate West, 5 Rathdown Walk, LISBURN, BT28 2RF. REC reference: 23/NI/0166, Protocol number: EDGE 161548, IRAS project ID: 328987 (December 2023).

## Results

Interrogation of the WHO Books for the presence of osteoclast-like cells in soft tissue tumours revealed 14 diagnoses. An additional 29 entities were extracted from the literature, including three recently described diagnoses [42–44], giving a total of 43 of 164 tumours (Supplementary Table S1).

### Mineralisation in soft tissue tumours

**FIGURE 2:**
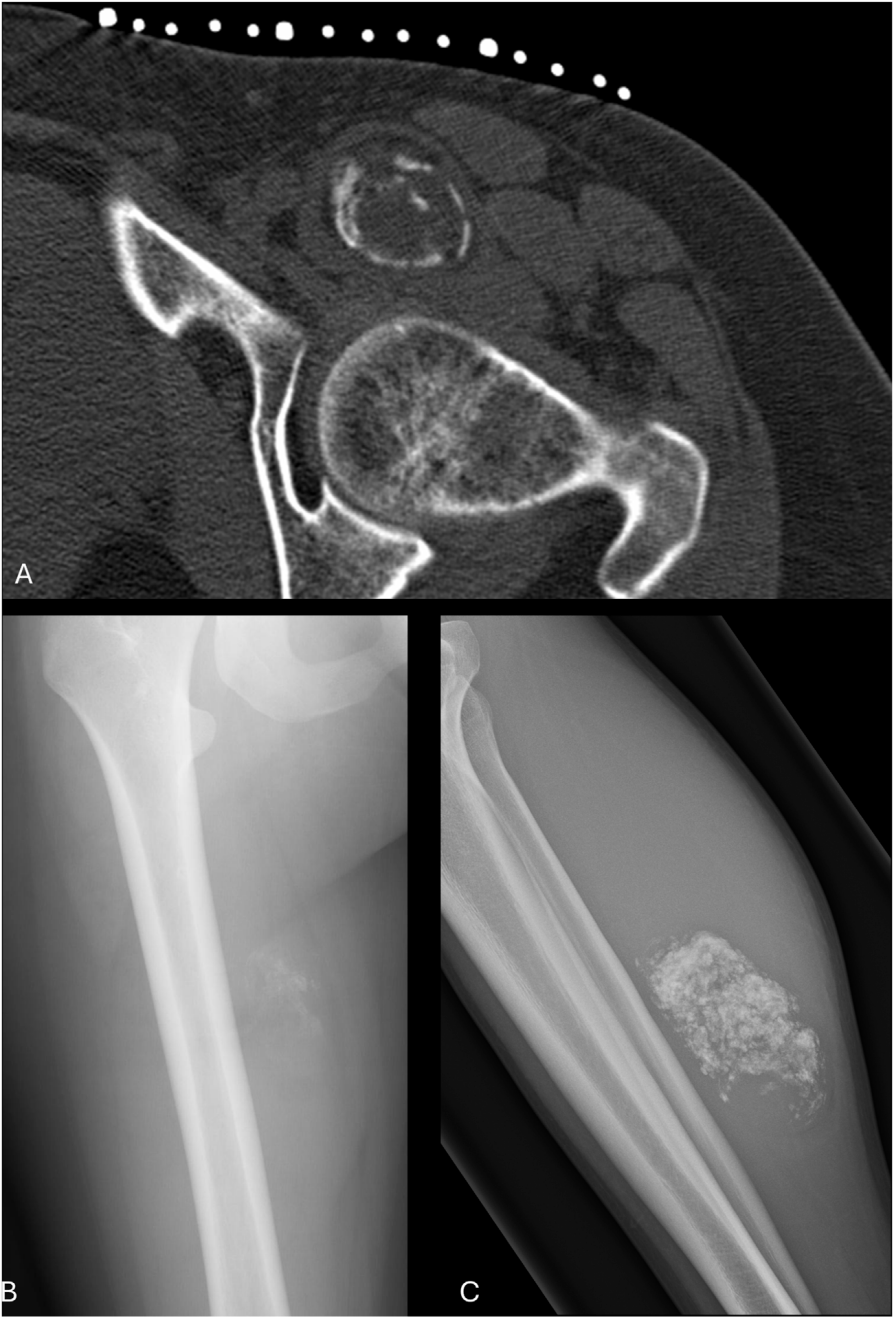
Radiology of calcified soft tissue masses. Axial CT shows an ossifying fibromyxoid tumour anterior to the left hip with a thin, incomplete bone margin **(A)**; AP radiograph of the right femur shows faint amorphous mineralisation projected medial to the bone in a synovial sarcoma **(B)**; lateral radiograph of the right tibia and fibula shows dense mineralisation in the posterior calf in a low-grade fibromyxoid sarcoma **(C)**. Each case shown harboured the relevant recurrent fusion gene characteristic of the tumour type namely PHF1::HCFC1, SS18-SSX and FUS::CREB3L2.

**FIGURE 3:**
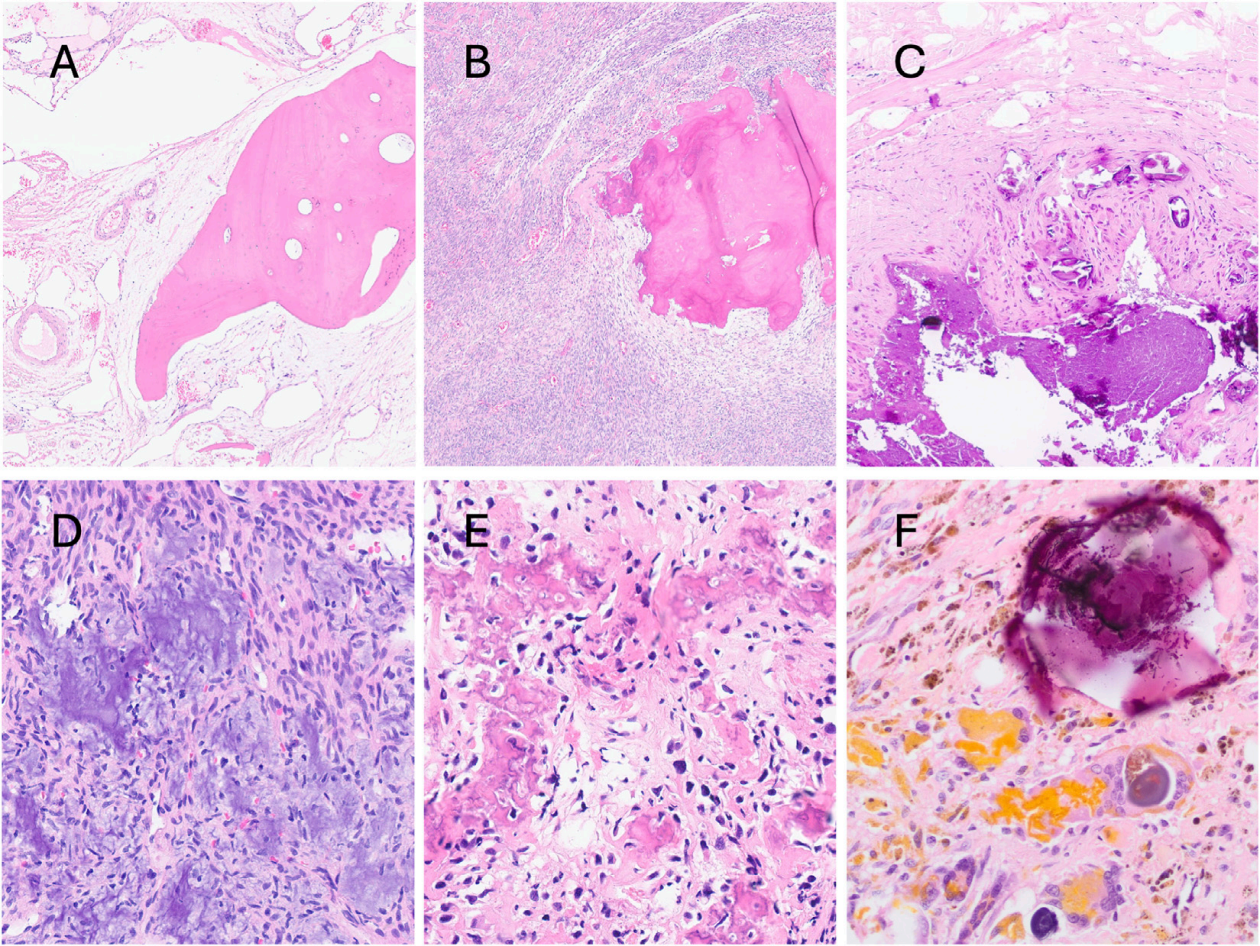
Photomicrographs of hematoxylin and eosin-stained sections of soft tissue lesions exhibiting different forms of mineralisation. Metaplastic mature compact bone in an intramuscular vascular malformation **(A)**. Calcification in synovial sarcoma **(B)**; tumoral calcinosis **(C)**; grungy calcifications in phosphaturic mesenchymal tumour **(D)**; irregular bone deposition in extraskeletal osteosarcoma **(E)**; psammoma bodies in malignant melanotic nerve sheath tumour **(F)**.

Next, we performed a literature search for mineralisation in soft tissue tumours. This revealed that 69 of the 164 tumours identified above contained mineralisation, 30 of which were also reported to harbour osteoclasts (Supplementary Table S1). However, in most of these lesions, the amount of mineralisation was limited and present infrequently and importantly, the feature was not useful for prompting a diagnosis. Conversely, mineralisation is an essential diagnostic requirement for some soft tissue tumours, for example, extra-skeletal osteosarcoma in which osteoclast-like cells can frequently be present [45] (Table 1). Finally, the search revealed sarcomas in which mineralisation was not an infrequent occurrence, and in which osteoclast-like cells have not been reported, but where it is a useful diagnostic hint for both pathologists and radiologists. Synovial sarcoma and ossifying fibromyxoid tumour are good examples, with mineralisation reported in 30% and 67% of cases, respectively, although this is old literature and was not based on molecular classification [46, 48]. Less recognised mineralisation occurs in low grade fibromyxoid sarcoma [49] (Figure 2). Other examples are listed in Table 1. Figure 3 highlights different types of mineralisation that are found in soft tissue lesions.

**TABLE 1:** Soft tissue tumours in which mineralisation (ossification and or calcification) represents a common feature and or is diagnostically useful.

Diagnosis	Mineralisation
^a^Calcifying aponeurotic fibroma	Essential criteria
^a^Extra-skeletal osteosarcoma (and other sarcomas with osteosarcomatous differentiation e.g. malignant peripheral nerve sheath tumour and dedifferentiated liposarcoma)	Essential criteria
^a^Phosphaturic mesenchymal tumour	Essential criteria
^a^Myositis ossificans/fibro-osseous pseudo-tumour of the fingers	Essential criteria
^a^Soft tissue chondroma	Common (% n/a)
Ossifying fibromyxoid tumour	67% [46]
Malignant melanotic nerve sheath tumour	40% [47]
Synovial Sarcoma	30% [48]

^a^ In which osteoclast-like cells are commonly seen.

### Osteoclasts in soft tissue tumours

Of the 43 tumour types with osteoclast-like cells identified in the WHO Books and the literature most have only been reported as single case reports or small series (Supplementary Table S1). We found only six tumour types that were reported to frequently contain ectopic osteoclasts and in which their distribution is not restricted to the site where bone or calcified material is deposited (Table 2). Giant cell tumour of soft parts, for which osteoclast-like giant cells are an essential diagnostic requirement, but where focal bone deposition is reported in 40.1% of cases [55], tenosynovial giant cell tumours, which harbour osteoclasts in 50%–100% of cases [52, 53], in 63% of plexiform fibrohistiocytic tumour [54] and 50% of Gastrointestinal clear cell sarcoma/malignant gastrointestinal neuroectodermal tumour [33]. Furthermore, osteoclast-like cells are reported to be found consistently, but less frequently, in 24% of 25 cases of angiomatoid fibrous histiocytoma [51], and in 10% of nodular fasciitis in the largest series (272 cases) published so far [50]. However, despite the authors casting doubt on whether these cells were true osteoclasts, on review of their published photomicrographs, we are confident that the multinucleated cells represented osteoclast-like cells. It is also reassuring to find that Montgomery et al. had already recognised osteoclast-like cells in nodular fasciitis [56]. It is noteworthy that both reports were published prior to the discovery of *USP6* being rearranged recurrently in nodular fasciitis. Likewise, Maqbool et al. [51] published their findings on osteoclasts in angiomatoid fibrous histiocytomas, but information on the presence of a*EWSR1* rearrangement was not provided.

**TABLE 2:** In-house review of whole slide images of soft tissue tumours for osteoclast-like cells.

Diagnosis	Number of cases reviewed	Number of cases containing osteoclast-like cells (%)	Previous reports of osteoclast-like cells: Number studied (%)
Nodular fasciitis^a^	200	36 (18)	(10) [50]
Undifferentiated sarcoma^a^ pleomorphic and spindle cell sarcoma	261	39 (15)	Case reports^b^
Myxofibrosarcoma	250	0	Not reported
Epithelioid sarcoma^a^	68	6 (9)	Case reports^b^
Leiomyosarcoma (non-uterine)	201	5 (2.5)	Case reports^b^
Clear cell sarcoma	80	0	Not reported
Angiomatoid fibrous histiocytoma	40	7 (17.5)	(24) [51]
Tenosynovial giant cell tumour	Not reviewed as osteoclasts seen in vast majority of cases		(50–100) [52, 53]
Giant cell tumour of soft parts^a^	Not reviewed as osteoclasts essential for the diagnosis		(100)
Plexiform fibrohistiocytic tumour	Not reviewed (insufficient cases)		(63) [54]
Gastrointestinal clear cell sarcoma	Not reviewed (insufficient cases)		(50) [33]

^a^ Reported to have mineralisation uncommonly and generally in limited amounts.

^b^ See Supplementary Table S1.

### In house internal WSIs library review

We next conducted an internal review of our WSI library for our Sarcoma AI project for some those entities identified in the literature to contain ectopic osteoclasts, generally in the absence of mineralisation, and listed in Table 2. In addition to the six diagnoses for which there is strong published evidence for osteoclast-like cells, we extended our review of WSI to an additional four diagnoses based on our observations (unpublished data) (Table 2).

**FIGURE 4:**
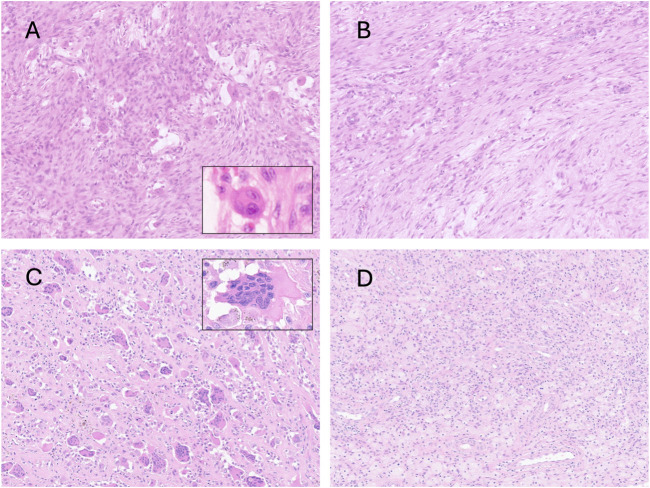
Photomicrographs of hematoxylin and eosin-stained slides showing variability in the distribution of osteoclast-like cells. Zonal variability in the same case of nodular fasciitis **(A,B)**; Two cases of tenosynovial giant cell tumour, with **(C)** and without **(D)** osteoclast-like cells. The insets show variability between the size of the cells and the number of nuclei can vary considerably.

The number, size and distribution of osteoclasts-like giant cells varied considerably between tumours but in general, they occurred scattered irregularly in clusters but sometimes formed sheets or occurred as individual cells. The number of nuclei in the osteoclasts also varied considerably (Figure 4).

Table 2 shows the tumour types in which we identified the frequency of osteoclast-like cells compared with previous reports. We have added to the literature by showing that 15% of undifferentiated sarcomas contained osteoclast-like cells. We also reviewed WSI from 250 myxofibrosarcomas, as these tumours often lose their myxoid matrix as the tumour evolves, and myxoid-poor areas can be difficult to distinguish from undifferentiated sarcomas histologically and genomically [57]. It was noteworthy that osteoclasts were not identified in any of the reviewed cases. However, large Touton-type giant cells were noted in these and some other tumour type, in addition to tumour giant cells (Figure 1aB,C).

**FIGURE 5:**
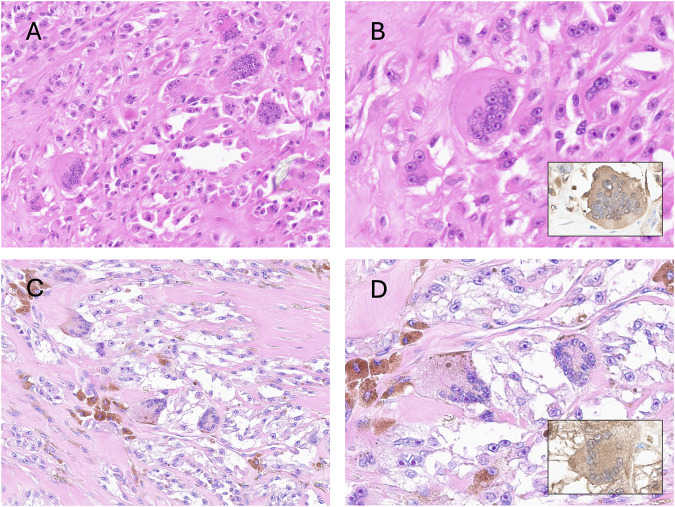
Photomicrographs of hematoxylin and eosin-stained sections showing two cases of clear cell sarcoma of tendons and aponeurosis. The top row case **(A,B)** contains tumoral giant cells with morphological features overlapping those of osteoclasts. The second case **(C,D)** contains tumour giant cells with wreath-like peripheral distribution, and melanin pigment is noted in the cytoplasm. The insets show immunoreactivity for S100.

Osteoclast-like cells are described as a prevalent feature of clear cell sarcoma of the gastrointestinal tract/malignant neuroectodermal tumours [58]. We were unable to provide information as to how commonly this is seen, as our image library contains insufficient cases. However, we reviewed 80 WSIs of clear cell sarcoma of soft tissue, a tumour with strong morphological and molecular similarities to its gastrointestinal counterpart and failed to identify osteoclast-like cells therein. Interestingly, tumour giant cells, which could be mistaken for osteoclast-like giant cells, were frequently encountered. These generally exhibit a peripheral nuclear distribution, but the cytoplasmic features are comparable to those of adjacent tumour cells (Figure 5).

## Discussion

Here we present a comprehensive overview of osteoclast-like cells and mineralisation in soft tissue tumours. Although not specific for any entity, awareness of these unusual features can help pathologists hone down on a differential diagnosis, reduce the number of molecular tests and render a definitive diagnosis more rapidly. At the outset, our focus was to catalogue tumours in which osteoclast-like cells occurred outside the skeletal system, such as in undifferentiated sarcomas, as we found it intriguing as to why this might occur.

However, we quickly realised that a significant number of soft tissue tumours are partly mineralised and therefore osteoclast-like cells would also be found in these lesions, as this is the physiological microenvironment to which they are recruited. Such examples include extra-skeletal osteosarcoma, dedifferentiated liposarcoma with osteosarcomatous differentiation and calcifying aponeurotic fibroma. Somewhat misleading from the nomenclature, only 67% of ossifying fibromyxoid tumours contain mineralisation [46]. Ultimately, we identified nine tumour types in which osteoclast-like cells appear relatively commonly in which they are not spatially related to mineralised tissue.

For pathologists to use histological features optimally for guiding clinical management requires that they interpret them in the clinical context. However, today, pathologists can often resolve a differential diagnosis by using molecular tests which screen for a wide range of alterations and provide a pathognomonic alteration. However, globally, most pathologists have limited access to sophisticated investigations and still rely on microscopy to make diagnoses and guide treatment. This highlights the importance of awareness of morphological features. As the WHO Classification of Tumours series of books represent a cornerstone for supporting pathologists world-wide in providing a diagnostic service, it remains important to provide diagnostically useful histological descriptions.

The value of recognising microscopic features but also reflecting on how they inform a diagnosis, has long provided insight into the pathogenesis of disease and has been the initiating stimulus that has resulted in the development of personalised treatment. For instance, recognition by pathologists of a gastrointestinal tract tumour that was neither of smooth muscle nor of nerve sheath origin and that is now recognised as “gastrointestinal stromal tumour” and is now treated with c-kit inhibitors has changed the clinical management and outcome of this disease [59]. Involving pathologists in discovery research has also played a crucial role in translational research and targeted treatments [5]; David Lacey, a pathologist with knowledge of bone disease and osteoclast biology, and his co-authors, recognised the importance of the profound osteopetrosis found in transgenic mice generated by the hepatic expression of an orphan protein identified while undertaking a sequencing study involving fetal rat intestine. They theorised that this molecule blocked osteoclast formation and the experiments led to the identification of Osteoprotegerin [60] and subsequently to the discovery of RANKL [55], and ultimately to the development of denosumab [61].

Although the cellular interactions at a molecular level involved in bone biology have changed dramatically since the discovery of RANKL [62], there is still much to learn. Research continues to reveal genetic alterations that disrupt the physiological process, including *H3F3A* mutations in giant cell tumour of bone [63], and germline mutations in the zinc finger protein 687 (*ZNF687*) and *PFN1* genes both resulting in multiple giant cell tumour-like lesions arising on a background of Pagetic bone disease [64–66]. However, the mechanisms by which these alterations drive disease is not understood. The availability of spatial transcriptomics now permits these questions to be addressed in an unprecedented manner by allowing the cellular interactions in tumours and their spatial relationships to be studied. This technology should allow identification of molecules involved in ectopic osteoclast recruitment in soft tissue tumours and provide new knowledge of both the physiological and pathological processes in bone and possibly deliver opportunities for the development of novel therapies.

There are limitations of the study: we only reviewed a maximum of two slides per case for the presence of osteoclasts. This is likely to have resulted in an under-estimate of the incidence of osteoclast-like cells in tumours analysed. Despite efforts to ensure that our literature searches were thorough, it is possible that we have overlooked some evidence. Review of the literature also demonstrated that some of the publications available on the subjects discussed are old, and employ historic nomenclature, such as malignant fibrous histiocytoma [21] and pigmented villonodular synovitis [18]. Furthermore, in some instances, the only literature available was prior or in the absence of robust molecular markers. Specifically, the incidence of mineralisation in synovial sarcoma is 30 years old and is based on radiological images [48].

The speed at which new information is being generated in all aspects of pathology is unprecedented. The challenge is how best to exploit it and use it safely. Harnessing artificial intelligence is likely to be the solution, but this will only be achieved safely with significant input from pathologists who appreciate and value morphological features [31].

## Data Availability

The original contributions presented in the study are included in the article/Supplementary Material, further inquiries can be directed to the corresponding author.
