# Supplementary material for: The value of morphology: osteoclast-like cells in soft tissue tumours
Source: Pathol Oncol Res. 2025 Sep 12;31:1612175. doi: 10.3389/pore.2025.1612175 (PMC12463735; doi:10.3389/pore.2025.1612175)
Supplement: Supplementary file 1 [file Table1.docx]

**Supplementary Table S1**

*Evidence of osteoclast-like cells and mineralisation processes in soft tissue tumours.*

| **WHO Book** | **Soft tissue tumours** | **Osteoclasts documented (WHO, +/- Ref.)** | **Mineralisation documented (WHO, +/- Ref.)** |
| --- | --- | --- | --- |
|  |  |  |  |
| Soft tissue and bone tumours (5th ed.) | Lipoma | No | WHO, [1] |
| Soft tissue and bone tumours (5th ed.) | Lipomatosis | No | No |
| Soft tissue and bone tumours (5th ed.) | Lipomatosis of nerve | No | No |
| Soft tissue and bone tumours (5th ed.) | Lipoblastoma and lipoblastomatosis | No | WHO, [2–4] |
| Soft tissue and bone tumours (5th ed.) | Angiolipoma | No | No |
| Soft tissue and bone tumours (5th ed.) | Myolipoma of soft tissue | No | Ref. [5] |
| Soft tissue and bone tumours (5th ed.) | Chondroid lipoma | WHO, [6] | WHO, [7] |
| Soft tissue and bone tumours (5th ed.) | Spindle cell lipoma and pleomorphic lipoma | No | WHO, [8] |
| Soft tissue and bone tumours (5th ed.) | Hibernoma | No | No |
| Soft tissue and bone tumours (5th ed.) | Atypical spindle cell/pleomorphic lipomatous tumour | No | WHO, [9] |
| Soft tissue and bone tumours (5th ed.) | Atypical lipomatous tumour/well-differentiated liposarcoma | No | WHO, [10, 11] |
| Soft tissue and bone tumours (5th ed.) | Dedifferentiated liposarcoma | Ref. [12] | WHO, [13] |
| Soft tissue and bone tumours (5th ed.) | Myxoid liposarcoma | No | WHO, [14] |
| Soft tissue and bone tumours (5th ed.) | Pleomorphic liposarcoma | No | No |
| Soft tissue and bone tumours (5th ed.) | Myxoid pleomorphic liposarcoma | No | No |
| Soft tissue and bone tumours (5th ed.) | Nodular fasciitis | WHO, [15, 16] | WHO, [17, 18] |
| Soft tissue and bone tumours (5th ed.) | Proliferative fasciitis and proliferative myositis | No | WHO, [19, 20] |
| Soft tissue and bone tumours (5th ed.) | Myositis ossificans and fibro-osseous pseudotumour of digits | WHO, [21] | WHO, [21] |
| Soft tissue and bone tumours (5th ed.) | Ischaemic fasciitis | No | No |
| Soft tissue and bone tumours (5th ed.) | Elastofibroma | No | No |
| Soft tissue and bone tumours (5th ed.) | Fibrous hamartoma of infancy | No | No |
| Soft tissue and bone tumours (5th ed.) | Fibromatosis colli | No | No |
| Soft tissue and bone tumours (5th ed.) | Juvenile hyaline fibromatosis | No | No |
| Soft tissue and bone tumours (5th ed.) | Inclusion body fibromatosis | No | No |
| Soft tissue and bone tumours (5th ed.) | Fibroma of tendon sheath | Ref. [22] | WHO |
| Soft tissue and bone tumours (5th ed.) | Desmoplastic fibroblastoma | No | No |
| Soft tissue and bone tumours (5th ed.) | Myofibroblastoma | No | No |
| Soft tissue and bone tumours (5th ed.) | Calcifying aponeurotic fibroma | WHO, [23] | WHO, [23] |
| Soft tissue and bone tumours (5th ed.) | EWSR1-SMAD3-positive fibroblastic tumour (emerging) | No | WHO, [24] |
| Soft tissue and bone tumours (5th ed.) | Angiomyofibroblastoma | No | No |
| Soft tissue and bone tumours (5th ed.) | Cellular angiofibroma | No | No |
| Soft tissue and bone tumours (5th ed.) | Angiofibroma of soft tissue | No | No |
| Soft tissue and bone tumours (5th ed.) | Nuchal-type fibroma | No | No |
| Soft tissue and bone tumours (5th ed.) | Acral fibromyxoma | No | WHO, [25] |
| Soft tissue and bone tumours (5th ed.) | Gardner fibroma | No | No |
| Soft tissue and bone tumours (5th ed.) | Palmar fibromatosis and plantar fibromatosis | No | WHO, [26] |
| Soft tissue and bone tumours (5th ed.) | Desmoid fibromatosis | No | No |
| Soft tissue and bone tumours (5th ed.) | Lipofibromatosis | No | WHO, [27] |
| Soft tissue and bone tumours (5th ed.) | Giant cell fibroblastoma | Ref. [28] | No |
| Soft tissue and bone tumours (5th ed.) | Dermatofibrosarcoma protuberans | No | No |
| Soft tissue and bone tumours (5th ed.) | Solitary fibrous tumour | No | WHO, [29] |
| Soft tissue and bone tumours (5th ed.) | Inflammatory myofibroblastic tumour | Ref. [30] | WHO, [31, 32] |
| Soft tissue and bone tumours (5th ed.) | Low-grade myofibroblastic sarcoma | Ref. [33] | Ref. [34] |
| Soft tissue and bone tumours (5th ed.) | Superficial CD34-positive fibroblastic tumour | No | No |
| Soft tissue and bone tumours (5th ed.) | Myxoinflammatory fibroblastic sarcoma | Ref. [35] | Ref. [36] |
| Soft tissue and bone tumours (5th ed.) | Infantile fibrosarcoma | No | No |
| Soft tissue and bone tumours (5th ed.) | Adult fibrosarcoma | No | No |
| Soft tissue and bone tumours (5th ed.) | Myxofibrosarcoma | No | No |
| Soft tissue and bone tumours (5th ed.) | Low-grade fibromyxoid sarcoma | No | WHO, [37, 38] |
| Soft tissue and bone tumours (5th ed.) | Sclerosing epithelioid fibrosarcoma | No | WHO, [39] |
| Soft tissue and bone tumours (5th ed.) | Tenosynovial giant cell tumour | WHO, [40–43] | No |
| Soft tissue and bone tumours (5th ed.) | Fibrous histiocytoma (dermatofibroma) | Ref. [44, 45] | No |
| Soft tissue and bone tumours (5th ed.) | Plexiform fibrohistiocytic tumour* | WHO, [46] | WHO, [47] |
| Soft tissue and bone tumours (5th ed.) | Giant cell tumour of soft tissue | WHO, [48] | WHO, [48] |
| Soft tissue and bone tumours (5th ed.) | Synovial haemangioma | No | No |
| Soft tissue and bone tumours (5th ed.) | Intramuscular angioma | No | No |
| Soft tissue and bone tumours (5th ed.) | Arteriovenous malformation/haemangioma | No | WHO |
| Soft tissue and bone tumours (5th ed.) | Venous haemangioma | No | WHO, |
| Soft tissue and bone tumours (5th ed.) | Anastomosing haemangioma | No | No |
| Soft tissue and bone tumours (5th ed.) | Epithelioid haemangioma | No | No |
| Soft tissue and bone tumours (5th ed.) | Lymphangioma and lymphangiomatosis | No | No |
| Soft tissue and bone tumours (5th ed.) | Tufted angioma and kaposiform haemangioendothelioma | No | No |
| Soft tissue and bone tumours (5th ed.) | Retiform haemangioendothelioma | No | No |
| Soft tissue and bone tumours (5th ed.) | Papillary intralymphatic angioendothelioma | No | No |
| Soft tissue and bone tumours (5th ed.) | Composite haemangioendothelioma | No | No |
| Soft tissue and bone tumours (5th ed.) | Kaposi sarcoma | No | No |
| Soft tissue and bone tumours (5th ed.) | Pseudomyogenic haemangioendothelioma | No | No |
| Soft tissue and bone tumours (5th ed.) | Epithelioid haemangioendothelioma | Ref. [49, 50] | WHO, [51] |
| Soft tissue and bone tumours (5th ed.) | Angiosarcoma | Ref. [52, 53] | Ref. [54] |
| Soft tissue and bone tumours (5th ed.) | Glomus tumour | No | No |
| Soft tissue and bone tumours (5th ed.) | Myopericytoma, including myofibroma | Ref. [55] | WHO, [56, 57] |
| Soft tissue and bone tumours (5th ed.) | Angioleiomyoma | No | WHO, [58] |
| Soft tissue and bone tumours (5th ed.) | Leiomyoma | Ref. [59] | WHO, [60, 61] |
| Soft tissue and bone tumours (5th ed.) | EBV-associated smooth muscle tumour | No | No |
| Soft tissue and bone tumours (5th ed.) | Inflammatory leiomyosarcoma | No | WHO |
| Soft tissue and bone tumours (5th ed.) | Leiomyosarcoma | WHO, [62–66] | WHO, [67, 68] |
| Soft tissue and bone tumours (5th ed.) | Rhabdomyoma | No | No |
| Soft tissue and bone tumours (5th ed.) | Embryonal rhabdomyosarcoma | No | No |
| Soft tissue and bone tumours (5th ed.) | Alveolar rhabdomyosarcoma | No | No |
| Soft tissue and bone tumours (5th ed.) | Pleomorphic rhabdomyosarcoma | Ref. [69] | Ref. [69] |
| Soft tissue and bone tumours (5th ed.) | Spindle cell / sclerosing rhabdomyosarcoma | No | No |
| Soft tissue and bone tumours (5th ed.) | Ectomesenchymoma | No | No |
| Soft tissue and bone tumours (5th ed.) | Gastrointestinal stromal tumour | Ref. [66, 70, 71] | WHO, [70] |
| Soft tissue and bone tumours (5th ed.) | Soft tissue chondroma | Ref. [72, 73] | WHO, [72] |
| Soft tissue and bone tumours (5th ed.) | Extraskeletal osteosarcoma | WHO, [74] | WHO, [74] |
| Soft tissue and bone tumours (5th ed.) | Schwannoma | No | Ref. [75] |
| Soft tissue and bone tumours (5th ed.) | Neurofibroma | No | WHO, [76] |
| Soft tissue and bone tumours (5th ed.) | Perineurioma | No | Ref. [77, 78] |
| Soft tissue and bone tumours (5th ed.) | Granular cell tumour | No | No |
| Soft tissue and bone tumours (5th ed.) | Dermal nerve sheath myxoma | No | No |
| Soft tissue and bone tumours (5th ed.) | Solitary circumscribed neuroma | No | No |
| Soft tissue and bone tumours (5th ed.) | Ectopic meningioma and meningothelial hamartoma | Ref. [79] | WHO, [80] |
| Soft tissue and bone tumours (5th ed.) | Benign triton tumour / neuromuscular choristoma | No | No |
| Soft tissue and bone tumours (5th ed.) | Hybrid nerve sheath tumour | No | No |
| Soft tissue and bone tumours (5th ed.) | Malignant peripheral nerve sheath tumour | No | WHO, [81] |
| Soft tissue and bone tumours (5th ed.) | Malignant melanotic nerve sheath tumour | Ref. [82] | WHO, [82, 83] |
| Soft tissue and bone tumours (5th ed.) | Intramuscular myxoma | No | No |
| Soft tissue and bone tumours (5th ed.) | Juxta-articular myxoma | No | No |
| Soft tissue and bone tumours (5th ed.) | Deep (aggressive) angiomyxoma | No | No |
| Soft tissue and bone tumours (5th ed.) | Angiomatoid fibrous histiocytoma | Ref. [84] | No |
| Soft tissue and bone tumours (5th ed.) | Ossifying fibromyxoid tumour | No | WHO, [85] |
| Soft tissue and bone tumours (5th ed.) | Myoepithelioma, myoepithelial carcinoma, and mixed tumour | Ref. [86] | WHO, [87] |
| Soft tissue and bone tumours (5th ed.) | Pleomorphic hyalinizing angiectatic tumour of soft parts | No | No |
| Soft tissue and bone tumours (5th ed.) | Haemosiderotic fibrolipomatous tumour | WHO, [88] | Ref. [89] |
| Soft tissue and bone tumours (5th ed.) | Phosphaturic mesenchymal tumour | WHO, [90] | WHO, [90] |
| Soft tissue and bone tumours (5th ed.) | NTRK-rearranged spindle cell neoplasm (emerging) | Ref. [91] | No |
| Soft tissue and bone tumours (5th ed.) | Atypical fibroxantoma | WHO, [92, 93] | No |
| Soft tissue and bone tumours (5th ed.) | Synovial sarcoma | No | WHO, [94] |
| Soft tissue and bone tumours (5th ed.) | Epithelioid sarcoma | Ref. [95] | WHO, [96] |
| Soft tissue and bone tumours (5th ed.) | Alveolar soft part sarcoma | No | WHO |
| Soft tissue and bone tumours (5th ed.) | Clear cell sarcoma of soft tissue | No | No |
| Soft tissue and bone tumours (5th ed.) | Extraskeletal myxoid chondrosarcoma | No | No |
| Soft tissue and bone tumours (5th ed.) | Desmoplastic small round cell tumour | No | Ref. [97] |
| Soft tissue and bone tumours (5th ed.) | Extrarenal rhabdoid tumour | Ref. [98] | No |
| Soft tissue and bone tumours (5th ed.) | PEComa | No | Ref. [99] |
| Soft tissue and bone tumours (5th ed.) | Intimal sarcoma | Ref. [100] | WHO, [101] |
| Soft tissue and bone tumours (5th ed.) | Undifferentiated sarcoma | Ref. [102–105] | No |
| Eye and Orbit tumours (5th ed.) | Conjunctival stromal tumour and ocular surface fibroma | No | No |
| Skin tumours (5th ed.) | Sclerotic fibroma | No | No |
| Skin tumours (5th ed.) | Pleomorphic fibroma | No | No |
| Skin tumours (5th ed.) | Fibrous papule | No | No |
| Skin tumours (5th ed.) | Fibroblastic connective tissue naevus | No | No |
| Skin tumours (5th ed.) | Cutaneous myxoma (superficial angiomyxoma) | No | No |
| Skin tumours (5th ed.) | Multinucleate cell angiohistiocytoma | No | No |
| Skin tumours (5th ed.) | Cutaneous epithelioid angiomatous nodule | No | No |
| Skin tumours (5th ed.) | Atypical intradermal smooth muscle neoplasm | Ref. [106] | No |
| Skin tumours (5th ed.) | Dermal hyperneury/Epithelial sheath neuroma | No | No |
| Skin tumours (5th ed.) | Cellular neurothekeoma | No | WHO, [107] |
| Skin tumours (5th ed.) | Epithelioid fibrous histiocytoma | No | WHO, [108] |
| Skin tumours (5th ed.) | Non-neural granular cell tumour | No | No |
| Skin tumours (5th ed.) | Pleomorphic dermal sarcoma | WHO, [109] | No |
| Skin tumours (5th ed.) | CRTC1::TRIM11 cutaneous tumour | No | No |
| Hematolymphoid tumours (5th ed.) | Intranodal palisaded myofibroblastoma | No | No |
| Thoracic tumours (5th ed.) | Pleuropulmonary blastoma | No | No |
| Thoracic tumours (5th ed.) | Congenital peribronchial myofibroblastic tumour | No | No |
| Thoracic tumours (5th ed.) | Primary pulmonary myxoid sarcoma with EWSR1-CREB1 fusion | No | No |
| Head and Neck tumours (5th ed.) | Biphenotipic sinonasal sarcoma | No | No |
| Head and Neck tumours (5th ed.) | Ectomesenchymal chondromyxoid tumor | No | No |
| Head and Neck tumours (5th ed.) | GLI1-altered soft tissue tumours | No | Ref. [110] |
| Urinary and Male Genital Tumours (5th ed.) | Renal Haemangioblastoma | No | Ref. [111] |
| Urinary and Male Genital Tumours (5th ed.) | Juxtaglomerular cell tumour | No | Ref. [112] |
| Urinary and Male Genital Tumours (5th ed.) | Renomedullary interstitial cell tumour | No | No |
| Urinary and Male Genital Tumours (5th ed.) | Ossifying renal tumour of infancy | No | WHO, [113] |
| Urinary and Male Genital Tumours (5th ed.) | Congenital mesoblastic nephroma | No | No |
| Urinary and Male Genital Tumours (5th ed.) | Rhabdoid tumour of the kidney | No | No |
| Urinary and Male Genital Tumours (5th ed.) | Clear cell sarcoma of the kidney | No | No |
| Urinary and Male Genital Tumours (5th ed.) | Prostatic stromal tumour of uncertain malignant potential | No | No |
| Urinary and Male Genital Tumours (5th ed.) | Prostatic stromal sarcoma | No | No |
| Urinary and Male Genital Tumours (5th ed.) | Myointimoma | No | No |
| Breast tumours (5th ed.) | Atypical vascular lesion of breast | No | No |
| Breast tumours (5th ed.) | Fibroadenoma of breast | Ref. [114] | WHO, [115] |
| Breast tumours (5th ed.) | Phyllodes tumour | Ref. [116, 117] | WHO, [118, 119] |
| Dygestive System Tumours (5th ed.) | Inflammatory fibroid polyp | No | No |
| Dygestive System Tumours (5th ed.) | Plexiform fibromyxoma | No | No |
| Dygestive System Tumours (5th ed.) | Mesenchymal hamartoma of the liver | No | No |
| Dygestive System Tumours (5th ed.) | Calciyfing nested stromal-epithelial tumour of the liver | No | WHO, [120] |
| Dygestive System Tumours (5th ed.) | Gastrointestinal clear cell sarcoma/ malignant gastrointestinal neuroectodermal tumour | WHO, [121] | No |
| n/a | NUTM1-rearranged colorectal sarcoma | Ref. [122] | No |
| n/a | Calcified chondroid mesenchymal neoplasm with FN1-receptor tyrosine kinase gene fusions | No | Ref. [123] |
| n/a | Inflammatory rhabdomyoblastic tumour | No | Ref. [124] |
| n/a | Pseudoendocrine sarcoma | No | Ref. [125] |
| n/a | Low-grade spindle cell sarcoma with storiform pattern and EWSR1/FUS-NACC1 fusions | No | Ref. [126] |
| n/a | Clear cell stromal tumour of the lung | Ref. [127] | Ref. [127] |
| n/a | Xanthogranulomatous epithelial tumour | Ref. [128] | No |

**References**

1. Fritchie KJ, Renner JB, Rao KW, Esther RJ. Osteolipoma: radiological, pathological, and cytogenetic analysis of three cases. *Skeletal Radiol* (2012) 41:237–244. doi: 10.1007/s00256-011-1241-0

2. Coffin CM, Lowichik A, Putnam A. Lipoblastoma (LPB): a clinicopathologic and immunohistochemical analysis of 59 cases. *Am J Surg Pathol* (2009) 33:1705–1712. doi: 10.1097/PAS.0b013e3181b76462

3. Collins MH, Chatten J. Lipoblastoma/lipoblastomatosis: a clinicopathologic study of 25 tumors. *Am J Surg Pathol* (1997) 21:1131–1137. doi: 10.1097/00000478-199710000-00002

4. Craver RD, Henrich S, Kao YS. Fibrous lipoblastoma with 8q11.2 abnormality. *Cancer Genet Cytogenet* (2006) 171:112–114. doi: 10.1016/j.cancergencyto.2006.06.020

5. Fukushima M, Schaefer I-M, Fletcher CDM. Myolipoma of Soft Tissue: Clinicopathologic Analysis of 34 Cases. *Am J Surg Pathol* (2017) 41:153–160. doi: 10.1097/PAS.0000000000000737

6. Alyousef M, Al Nemer A. Chondroid lipoma associated with osteoclast-like multinucleated giant cells - a case report. *APMIS* (2017) 125:506–508. doi: 10.1111/apm.12681

7. Hoch B, Hermann G, Klein MJ, Abdelwahab IF. Ossifying chondroid lipoma. *Skeletal Radiol* (2008) 37:475–480. doi: 10.1007/s00256-007-0444-x

8. Fletcher CD, Martin-Bates E. Spindle cell lipoma: a clinicopathological study with some original observations. *Histopathology* (1987) 11:803–817. doi: 10.1111/j.1365-2559.1987.tb01884.x

9. Mariño-Enriquez A, Nascimento AF, Ligon AH, Liang C, Fletcher CDM. Atypical Spindle Cell Lipomatous Tumor: Clinicopathologic Characterization of 232 Cases Demonstrating a Morphologic Spectrum. *Am J Surg Pathol* (2017) 41:234–244. doi: 10.1097/PAS.0000000000000770

10. Yoshida A, Ushiku T, Motoi T, Shibata T, Fukayama M, Tsuda H. Well-differentiated liposarcoma with low-grade osteosarcomatous component: an underrecognized variant. *Am J Surg Pathol* (2010) 34:1361–1366. doi: 10.1097/PAS.0b013e3181ebcc45

11. Evans HL. Atypical lipomatous tumor, its variants, and its combined forms: a study of 61 cases, with a minimum follow-up of 10 years. *Am J Surg Pathol* (2007) 31:1–14. doi: 10.1097/01.pas.0000213406.95440.7a

12. Sosnowska-Sienkiewicz P, Mańkowski P, Stadnik H, Dłubak A, Czekała A, Karczewski M. A Rare Case of Dedifferentiated Liposarcoma with Osteosarcomatous Differentiation-Diagnostic and Therapeutic Challenges. *Diseases* (2023) 12:6. doi: 10.3390/diseases12010006

13. Yamashita K, Kohashi K, Yamada Y, Ishii T, Nishida Y, Urakawa H, et al. Osteogenic differentiation in dedifferentiated liposarcoma: a study of 36 cases in comparison to the cases without ossification. *Histopathology* (2018) 72:729–738. doi: 10.1111/his.13421

14. Weingertner N, Neuville A, Chibon F, Ray-Coquard I, Marcellin L, Ghnassia J-P. Myxoid liposarcoma with heterologous components: dedifferentiation or metaplasia? A FISH-documented and CGH-documented case report. *Appl Immunohistochem Mol Morphol* (2015) 23:230–235. doi: 10.1097/PAI.0000000000000008

15. Montgomery EA, Meis JM. Nodular fasciitis. Its morphologic spectrum and immunohistochemical profile. *Am J Surg Pathol* (1991) 15:942–948.

16. Lu L, Lao IW, Liu X, Yu L, Wang J. Nodular fasciitis: a retrospective study of 272 cases from China with clinicopathologic and radiologic correlation. *Ann Diagn Pathol* (2015) 19:180–185. doi: 10.1016/j.anndiagpath.2015.03.013

17. Kwittken J, Branche M. Fasciitis ossificans. *Am J Clin Pathol* (1969) 51:251–255. doi: 10.1093/ajcp/51.2.251

18. Daroca PJ, Pulitzer DR, LoCicero J. Ossifying fasciitis. *Arch Pathol Lab Med* (1982) 106:682–685.

19. Bensalah A, Elbouardi N, Douida A, Haloua M, Alami B, Boubbou M, et al. Proliferative myositis: case report and review of the literature. *Radiol Case Rep* (2021) 16:1902–1906. doi: 10.1016/j.radcr.2021.04.042

20. Chung EB, Enzinger FM. Proliferative fasciitis. *Cancer* (1975) 36:1450–1458. doi: 10.1002/1097-0142(197510)36:4<1450::aid-cncr2820360437>3.0.co;2-4

21. Sumiyoshi K, Tsuneyoshi M, Enjoji M. Myositis ossificans. A clinicopathologic study of 21 cases. *Acta Pathol Jpn* (1985) 35:1109–1122.

22. Pižem J, Matjašič A, Zupan A, Luzar B, Šekoranja D, Dimnik K. Fibroma of tendon sheath is defined by a USP6 gene fusion-morphologic and molecular reappraisal of the entity. *Mod Pathol* (2021) 34:1876–1888. doi: 10.1038/s41379-021-00836-4

23. Fetsch JF, Miettinen M. Calcifying aponeurotic fibroma: a clinicopathologic study of 22 cases arising in uncommon sites. *Hum Pathol* (1998) 29:1504–1510. doi: 10.1016/s0046-8177(98)90022-3

24. Kao Y-C, Flucke U, Eijkelenboom A, Zhang L, Sung Y-S, Suurmeijer AJH, et al. Novel EWSR1-SMAD3 Gene Fusions in a Group of Acral Fibroblastic Spindle Cell Neoplasms. *Am J Surg Pathol* (2018) 42:522–528. doi: 10.1097/PAS.0000000000001002

25. Avila C, Milani-Nejad N, Carr D. Superficial Acral Fibromyxoma with Ossification on the Fingertip. *J of Skin* (2020) 4:294–296. doi: 10.25251/skin.4.3.17

26. Sadideen H, Athanasou N, Ashmore A, McNab I. Heterotopic ossification in Dupuytren’s disease: clinical and histological significance. *J Bone Joint Surg Br* (2011) 93:1676–1678. doi: 10.1302/0301-620X.93B12.27438

27. Al-Ibraheemi A, Folpe AL, Perez-Atayde AR, Perry K, Hofvander J, Arbajian E, et al. Aberrant receptor tyrosine kinase signaling in lipofibromatosis: a clinicopathological and molecular genetic study of 20 cases. *Mod Pathol* (2019) 32:423–434. doi: 10.1038/s41379-018-0150-3

28. Hirose T, Sasaki M, Shintaku M, Hasegawa T, Kudo E, Sano T, et al. Giant cell fibroblastoma. A case report. *Acta Pathol Jpn* (1990) 40:540–544. doi: 10.1111/j.1440-1827.1990.tb01597.x

29. Collini P, Negri T, Barisella M, Palassini E, Tarantino E, Pastorino U, et al. High-grade sarcomatous overgrowth in solitary fibrous tumors: a clinicopathologic study of 10 cases. *Am J Surg Pathol* (2012) 36:1202–1215. doi: 10.1097/PAS.0b013e31825748f0

30. Cole B, Zhou H, McAllister N, Afify Z, Coffin CM. Inflammatory myofibroblastic tumor with thrombocytosis and a unique chromosomal translocation With ALK rearrangement. *Arch Pathol Lab Med* (2006) 130:1042–1045. doi: 10.5858/2006-130-1042-IMTWTA

31. Thompson LDR. Inflammatory Myofibroblastic Tumor. *Ear Nose Throat J* (2021) 100:520S-521S. doi: 10.1177/0145561319890165

32. Coffin CM, Watterson J, Priest JR, Dehner LP. Extrapulmonary inflammatory myofibroblastic tumor (inflammatory pseudotumor). A clinicopathologic and immunohistochemical study of 84 cases. *Am J Surg Pathol* (1995) 19:859–872. doi: 10.1097/00000478-199508000-00001

33. Fisher C. Myofibroblastic malignancies. *Adv Anat Pathol* (2004) 11:190–201. doi: 10.1097/01.pap.0000131773.16130.aa

34. Mentzel T, Dry S, Katenkamp D, Fletcher CD. Low-grade myofibroblastic sarcoma: analysis of 18 cases in the spectrum of myofibroblastic tumors. *Am J Surg Pathol* (1998) 22:1228–1238. doi: 10.1097/00000478-199810000-00008

35. Laskin WB, Fetsch JF, Miettinen M. Myxoinflammatory fibroblastic sarcoma: a clinicopathologic analysis of 104 cases, with emphasis on predictors of outcome. *Am J Surg Pathol* (2014) 38:1–12. doi: 10.1097/PAS.0b013e31829f3d85

36. Papke DJ, Odintsov I, Dickson BC, Nucci MR, Agaimy A, Fletcher CDM. Myxoid Inflammatory Myofibroblastic Sarcoma: Clinicopathologic Analysis of 25 Cases of a Distinctive Sarcoma With Deceptively Bland Morphology and Aggressive Clinical Behavior. *Am J Surg Pathol* (2024) 48:1005–1016. doi: 10.1097/PAS.0000000000002231

37. Guillou L, Benhattar J, Gengler C, Gallagher G, Ranchère-Vince D, Collin F, et al. Translocation-positive low-grade fibromyxoid sarcoma: clinicopathologic and molecular analysis of a series expanding the morphologic spectrum and suggesting potential relationship to sclerosing epithelioid fibrosarcoma: a study from the French Sarcoma Group. *Am J Surg Pathol* (2007) 31:1387–1402. doi: 10.1097/PAS.0b013e3180321959

38. Miyake M, Tateishi U, Maeda T, Arai Y, Seki K, Hasegawa T, et al. CT and MRI features of low-grade fibromyxoid sarcoma in the shoulder of a pediatric patient. *Radiat Med* (2006) 24:511–514. doi: 10.1007/s11604-006-0057-7

39. Meis-Kindblom JM, Kindblom LG, Enzinger FM. Sclerosing epithelioid fibrosarcoma. A variant of fibrosarcoma simulating carcinoma. *Am J Surg Pathol* (1995) 19:979–993. doi: 10.1097/00000478-199509000-00001

40. Monaghan H, Salter DM, Al-Nafussi A. Giant cell tumour of tendon sheath (localised nodular tenosynovitis): clinicopathological features of 71 cases. *J Clin Pathol* (2001) 54:404–407. doi: 10.1136/jcp.54.5.404

41. Ushijima M, Hashimoto H, Tsuneyoshi M, Enjoji M. Giant cell tumor of the tendon sheath (nodular tenosynovitis). A study of 207 cases to compare the large joint group with the common digit group. *Cancer* (1986) 57:875–884. doi: 10.1002/1097-0142(19860215)57:4<875::aid-cncr2820570432>3.0.co;2-y

42. Jones FE, Soule EH, Coventry MB. Fibrous xanthoma of synovium (giant-cell tumor of tendon sheath, pigmented nodular synovitis). A study of one hundred and eighteen cases. *J Bone Joint Surg Am* (1969) 51:76–86.

43. Somerhausen NS, Fletcher CD. Diffuse-type giant cell tumor: clinicopathologic and immunohistochemical analysis of 50 cases with extraarticular disease. *Am J Surg Pathol* (2000) 24:479–492. doi: 10.1097/00000478-200004000-00002

44. Kutchemeshgi M, Barr RJ, Henderson CD. Dermatofibroma with osteoclast-like giant cells. *Am J Dermatopathol* (1992) 14:397–401. doi: 10.1097/00000372-199210000-00004

45. González-Vilas D, García-Gavín J, Ginarte M, Rodríguez-Blanco I, Toribio J. Ulcerated dermatofibroma with osteoclast-like giant cells. *J Cutan Pathol* (2009) 36 Suppl 1:16–19. doi: 10.1111/j.1600-0560.2009.01195.x

46. Moosavi C, Jha P, Fanburg-Smith JC. An update on plexiform fibrohistiocytic tumor and addition of 66 new cases from the Armed Forces Institute of Pathology, in honor of Franz M. Enzinger, MD. *Ann Diagn Pathol* (2007) 11:313–319. doi: 10.1016/j.anndiagpath.2007.01.003

47. Thangaiah JJ, Dashti NK, Agaimy A, Fritchie K, Folpe AL. Plexiform fibrohistiocytic tumor: a clinicopathological and immunohistochemical study of 39 tumors, with evidence for a CSF1-producing “null cell” population. *Virchows Arch* (2022) 481:739–750. doi: 10.1007/s00428-022-03408-2

48. Oliveira AM, Dei Tos AP, Fletcher CD, Nascimento AG. Primary giant cell tumor of soft tissues: a study of 22 cases. *Am J Surg Pathol* (2000) 24:248–256. doi: 10.1097/00000478-200002000-00011

49. Tsuji M, Ozaki T, Tsutsumi A. Epithelioid hemangioendothelioma with osteoclast-like giant cells. *Pathol Res Pract* (2002) 198:501–505. doi: 10.1078/0344-0338-00293

50. Lamovec J, Sobel HJ, Zidar A, Jerman J. Epithelioid hemangioendothelioma of the anterior mediastinum with osteoclast-like giant cells. Light microscopic, immunohistochemical, and electron microscopic study. *Am J Clin Pathol* (1990) 93:813–817. doi: 10.1093/ajcp/93.6.813

51. Mentzel T, Beham A, Calonje E, Katenkamp D, Fletcher CD. Epithelioid hemangioendothelioma of skin and soft tissues: clinicopathologic and immunohistochemical study of 30 cases. *Am J Surg Pathol* (1997) 21:363–374. doi: 10.1097/00000478-199704000-00001

52. Belogrivtseva Y, Nobee A, Lazim A, Taraif S. Angiosarcoma with Osteoclast-Like Giant Cells: A Rare Presentation and Review of Literature. *American Journal of Clinical Pathology* (2023) 160:S6–S6. doi: 10.1093/ajcp/aqad150.013

53. Nawar NA, Olsen J, Jelic TM, He C. Primary Urinary Bladder Angiosarcoma with Osteoclast-Like Multinucleated Giant Cells: A Case Report and Literature Review. *Am J Case Rep* (2016) 17:143–149. doi: 10.12659/ajcr.896266

54. Meis-Kindblom JM, Kindblom LG. Angiosarcoma of soft tissue: a study of 80 cases. *Am J Surg Pathol* (1998) 22:683–697. doi: 10.1097/00000478-199806000-00005

55. Pižem J, Matjašič A, Zupan A, Luzar B, Šekoranja D, Dimnik K. Fibroma of tendon sheath is defined by a USP6 gene fusion-morphologic and molecular reappraisal of the entity. *Mod Pathol* (2021) 34:1876–1888. doi: 10.1038/s41379-021-00836-4

56. Herrtwich A, Sciot R, Ernst C. Myopericytoma in an Infant—Imaging Characteristics of a Rare but Benign Entity. *Journal of the Belgian Society of Radiology* (2024) 108:3. doi: 10.5334/jbsr.3441

57. Santo IDDO, Staziaki PV, Prilutskiy A, Sachs TE, Murakami AM. Solitary intramuscular myofibroma in an adult: Case report and MR imaging findings. *Clinical Imaging* (2020) 67:95–100. doi: 10.1016/j.clinimag.2020.05.027

58. Hachisuga T, Hashimoto H, Enjoji M. Angioleiomyoma. A clinicopathologic reappraisal of 562 cases. *Cancer* (1984) 54:126–130. doi: 10.1002/1097-0142(19840701)54:1<126::aid-cncr2820540125>3.0.co;2-f

59. Guilbert M-C, Samouëlian V, Rahimi K. Uterine Leiomyoma With Osteoclast-like Giant Cells. *Int J Gynecol Pathol* (2016) 35:30–32. doi: 10.1097/PGP.0000000000000204

60. Lu D, Chen H, Yang C, Liu Y, Mo H, Zhang Y. Primary ovarian leiomyoma with calcification: A case report. *Medicine (Baltimore)* (2024) 103:e39122. doi: 10.1097/MD.0000000000039122

61. López-Barea F, Rodríguez-Peralto JL, Burgos E, González-López J. Calcified leiomyoma of deep soft tissue. Report of a case in childhood. *Virchows Arch* (1994) 425:217–220. doi: 10.1007/BF00230360

62. Gibbons CLMH, Sun SG, Vlychou M, Kliskey K, Lau YS, Sabokbar A, et al. Osteoclast-like cells in soft tissue leiomyosarcomas. *Virchows Arch* (2010) 456:317–323. doi: 10.1007/s00428-010-0882-z

63. Mentzel T, Calonje E, Fletcher CD. Leiomyosarcoma with prominent osteoclast-like giant cells. Analysis of eight cases closely mimicking the so-called giant cell variant of malignant fibrous histiocytoma. *Am J Surg Pathol* (1994) 18:258–265.

64. Xu Z, Gu J, Zhang S, Zhang Z, Fang W. Leiomyosarcoma with osteoclast-like (LMS-OGC) giant cells the breast: A report of a rare case. *Thorac Cancer* (2019) 10:2054–2056. doi: 10.1111/1759-7714.13190

65. Katoh M, Shigematsu H. Leiomyosarcoma of the heart and its pulmonary metastasis, both with prominent osteoclast‐like multinucleated giant cells expressing tartrate‐resistant acid phosphatase activity. *Pathology International* (1999) 49:74–78. doi: 10.1046/j.1440-1827.1999.00817.x

66. Insabato L, Di Vizio D, Ciancia G, Pettinato G, Tornillo L, Terracciano L. Malignant Gastrointestinal Leiomyosarcoma and Gastrointestinal Stromal Tumor With Prominent Osteoclast-like Giant Cells. *Archives of Pathology & Laboratory Medicine* (2004) 128:440–443. doi: 10.5858/2004-128-440-MGLAGS

67. Chen E, O’Connell F, Fletcher CDM. Dedifferentiated leiomyosarcoma: clinicopathological analysis of 18 cases. *Histopathology* (2011) 59:1135–1143. doi: 10.1111/j.1365-2559.2011.04070.x

68. Bush CH, Reith JD, Spanier SS. Mineralization in Musculoskeletal Leiomyosarcoma: Radiologic—Pathologic Correlation. *American Journal of Roentgenology* (2003) 180:109–113. doi: 10.2214/ajr.180.1.1800109

69. Paner GP, Gasilionis V, Hammadeh R. A retroperitoneal mass in an elderly woman. Pleomorphic rhabdomyosarcoma, classic variant, with reactive osteoclast-like giant cells. *Arch Pathol Lab Med* (2005) 129:703–705. doi: 10.5858/2005-129-0703-ARMIAE

70. Do I-G, Park CK, Kim K-M. Gastrointestinal stromal tumour with osteoclast-like giant cells and aneurysmal bone cyst-like features. *Pathology* (2009) 41:396–397. doi: 10.1080/00313020902886977

71. Leung KM, Wong S, Chow TC, Lee KC. A malignant gastrointestinal stromal tumor with osteoclast-like giant cells. *Arch Pathol Lab Med* (2002) 126:972–974. doi: 10.5858/2002-126-0972-AMGSTW

72. Chung EB, Enzinger FM. Chondroma of soft parts. *Cancer* (1978) 41:1414–1424. doi: 10.1002/1097-0142(197804)41:4<1414::aid-cncr2820410429>3.0.co;2-o

73. Cates JM, Rosenberg AE, O’Connell JX, Nielsen GP. Chondroblastoma-like chondroma of soft tissue: an underrecognized variant and its differential diagnosis. *Am J Surg Pathol* (2001) 25:661–666. doi: 10.1097/00000478-200105000-00015

74. Hesni S, Lindsay D, O’Donnell P, Saifuddin A. Extra-skeletal osteosarcoma: a review. *Skeletal Radiol* (2023) 52:633–648. doi: 10.1007/s00256-022-04193-4

75. Din NU, Fritchie K, Tariq MU, Ahmed A, Ahmad Z. Calcification and ossification in conventional schwannoma: A clinicopathologic study of 32 cases. *Neuropathology* (2020) 40:144–151. doi: 10.1111/neup.12622

76. Muroyama Y, Miura C, Imai Y, Suzuki T. Ossification of neurofibroma in neurofibromatosis type 1, a case report of a rare presentation. *Int J Surg Case Rep* (2024) 122:110151. doi: 10.1016/j.ijscr.2024.110151

77. Rank JP, Rostad SW. Perineurioma with ossification: a case report with immunohistochemical and ultrastructural studies. *Arch Pathol Lab Med* (1998) 122:366–370.

78. Hornick JL, Fletcher CDM. Soft tissue perineurioma: clinicopathologic analysis of 81 cases including those with atypical histologic features. *Am J Surg Pathol* (2005) 29:845–858. doi: 10.1097/01.pas.0000155166.86409.d2

79. Barresi V, Caffo M, Ieni A, Alafaci C, Tuccari G. Osteoblastic meningiomas: clinico-pathological and immunohistochemical features of an uncommon variant. *J Neurooncol* (2011) 105:225–232. doi: 10.1007/s11060-011-0588-3

80. Chotai SP, Mrak RE, Mutgi SA, Medhkour A. Ossification in an extra-intradural spinal meningioma-pathologic and surgical vistas. *Spine J* (2013) 13:e21-26. doi: 10.1016/j.spinee.2013.06.102

81. Ducatman BS, Scheithauer BW, Piepgras DG, Reiman HM, Ilstrup DM. Malignant peripheral nerve sheath tumors. A clinicopathologic study of 120 cases. *Cancer* (1986) 57:2006–2021. doi: 10.1002/1097-0142(19860515)57:10<2006::aid-cncr2820571022>3.0.co;2-6

82. Luzar B, Shanesmith R, Ramakrishnan R, Fisher C, Calonje E. Cutaneous epithelioid malignant peripheral nerve sheath tumour: a clinicopathological analysis of 11 cases. *Histopathology* (2016) 68:286–296. doi: 10.1111/his.12756

83. Torres-Mora J, Dry S, Li X, Binder S, Amin M, Folpe AL. Malignant melanotic schwannian tumor: a clinicopathologic, immunohistochemical, and gene expression profiling study of 40 cases, with a proposal for the reclassification of “melanotic schwannoma.” *Am J Surg Pathol* (2014) 38:94–105. doi: 10.1097/PAS.0b013e3182a0a150

84. Maqbool H, Bashir S, Hassan U, Hussain M, Mushtaq S, Ishtiaq S. Angiomatoid Fibrous Histiocytoma: A Tumor With Uncertain Behavior and Various Clinicopathological Presentations. *Cureus* (2022) 14:e28985. doi: 10.7759/cureus.28985

85. Folpe AL, Weiss SW. Ossifying fibromyxoid tumor of soft parts: a clinicopathologic study of 70 cases with emphasis on atypical and malignant variants. *Am J Surg Pathol* (2003) 27:421–431. doi: 10.1097/00000478-200304000-00001

86. Nagao T, Sugano I, Ishida Y, Tajima Y, Matsuzaki O, Konno A, et al. Salivary gland malignant myoepithelioma: a clinicopathologic and immunohistochemical study of ten cases. *Cancer* (1998) 83:1292–1299. doi: 10.1002/(sici)1097-0142(19981001)83:7<1292::aid-cncr4>3.0.co;2-l

87. Hornick JL, Fletcher CDM. Myoepithelial tumors of soft tissue: a clinicopathologic and immunohistochemical study of 101 cases with evaluation of prognostic parameters. *Am J Surg Pathol* (2003) 27:1183–1196. doi: 10.1097/00000478-200309000-00001

88. Browne T-J, Fletcher CDM. Haemosiderotic fibrolipomatous tumour (so-called haemosiderotic fibrohistiocytic lipomatous tumour): analysis of 13 new cases in support of a distinct entity. *Histopathology* (2006) 48:453–461. doi: 10.1111/j.1365-2559.2006.02360.x

89. West AT, Toms AP, Murphy J, Sultan M. Haemosiderotic fibrohistiocytic lipomatous lesion/tumour of the foot: MRI and histopathology. *Skeletal Radiol* (2007) 37:71–74. doi: 10.1007/s00256-007-0397-0

90. Folpe AL, Fanburg-Smith JC, Billings SD, Bisceglia M, Bertoni F, Cho JY, et al. Most osteomalacia-associated mesenchymal tumors are a single histopathologic entity: an analysis of 32 cases and a comprehensive review of the literature. *Am J Surg Pathol* (2004) 28:1–30. doi: 10.1097/00000478-200401000-00001

91. Grant L, Boyle W, Williams S, Pascoe J, Ganesan R. Uterine Neurotrophic Tyrosine Receptor Kinase Rearranged Spindle Cell Neoplasms: Three Cases of an Emerging Entity. *Int J Gynecol Pathol* (2024) 43:326–334. doi: 10.1097/PGP.0000000000000988

92. Anderson JD, Alhatem A, Li Y, Hurley MY. Cutaneous atypical fibroxanthoma with osteoclast‐like giant cell: A rare phenomenon. *J Cutan Pathol* (2022) 49:722–726. doi: 10.1111/cup.14229

93. Shaker N, Phelps R, Niedt G, Patel A, Wu D, Aung PP, et al. Cutaneous Atypical Fibroxanthoma With Osteoclast-Like Giant Cell: A Rare but Diagnostic Pitfall. *Am J Dermatopathol* (2023) 45:704–707. doi: 10.1097/DAD.0000000000002508

94. Milchgrub S, Ghandur-Mnaymneh L, Dorfman HD, Albores-Saavedra J. Synovial sarcoma with extensive osteoid and bone formation. *Am J Surg Pathol* (1993) 17:357–363. doi: 10.1097/00000478-199304000-00006

95. Rekhi B, Verma A, Jambhekar NA, Menon S, Laskar S, Merchant N, et al. Osteoclast-rich, proximal-type epithelioid sarcoma: clinicopathologic features of 3 unusual cases expanding the histomorphological spectrum. *Ann Diagn Pathol* (2016) 21:39–43. doi: 10.1016/j.anndiagpath.2015.12.003

96. Chase DR, Enzinger FM. Epithelioid sarcoma. Diagnosis, prognostic indicators, and treatment. *Am J Surg Pathol* (1985) 9:241–263.

97. Lae ME, Roche PC, Jin L, Lloyd RV, Nascimento AG. Desmoplastic small round cell tumor: a clinicopathologic, immunohistochemical, and molecular study of 32 tumors. *Am J Surg Pathol* (2002) 26:823–835. doi: 10.1097/00000478-200207000-00001

98. Coffin CM, Belchis D. “Immunohistology of Pediatric Neoplasms.,” *Diagnostic Immunohistochemistry*. Elsevier (2006). p. 611–636 doi: 10.1016/B978-0-443-06652-8.50021-1

99. Menéndez CL, Gil Ugarteburu R, Capilla Ampudia JM, Corte Torres G, Fuente E, Argüelles M. A case of pararenal PEComa with extensive bone metaplasia. *Virchows Arch* (2008) 452:349–350. doi: 10.1007/s00428-008-0581-1

100. Kamio T, Suko S, Tanoue K, Ichikado Y, Yoshida S, Hirayama T, et al. A case of sarcoma with osteoclast-like giant cells arising from pulmonary artery. *J Jpn Soc Clin Cytol* (2000) 39:20–25. doi: 10.5795/jjscc.39.20

101. Burke AP, Virmani R. Sarcomas of the great vessels. A clinicopathologic study. *Cancer* (1993) 71:1761–1773. doi: 10.1002/1097-0142(19930301)71:5<1761::aid-cncr2820710510>3.0.co;2-7

102. Flanagan AM, Chambers TJ. Osteoclasts are present in the giant cell variant of malignant fibrous histiocytoma. *The Journal of Pathology* (1989) 159:53–57. doi: 10.1002/path.1711590112

103. Singh B, Santos V, Guffin TN, Alexis R, Har-El G. Giant cell variant of malignant fibrous histiocytoma of the head and neck. *J Laryngol Otol* (1991) 105:1079–1081. doi: 10.1017/s0022215100118249

104. Srinivasamurthy BC, Kulandaivelu AR, Saha K, Saha A. Primary undifferentiated pleomorphic sarcoma of the breast in a young female: a case report. *World J Surg Oncol* (2016) 14:186. doi: 10.1186/s12957-016-0947-9

105. Balbi G, Di Martino L, Pitruzzella G, Pitruzzella D, Grauso F, Napolitano A, et al. Undifferentiated pleomorphic sarcoma with osteoclast-like giant cells of the female breast. *World J Surg Oncol* (2013) 11:21. doi: 10.1186/1477-7819-11-21

106. Sarma DP, Santos EE, Wang B. Leiomyosarcoma of the skin with osteoclast-like giant cells: a case report. *J Med Case Reports* (2007) 1:180. doi: 10.1186/1752-1947-1-180

107. Goette DK. Calcifying neurothekeoma. *J Dermatol Surg Oncol* (1986) 12:958–960. doi: 10.1111/j.1524-4725.1986.tb02137.x

108. Martinez AP, Zou Y, Billings SD, Folpe AL. “Chondroblastoma-like” epithelioid fibrous histiocytoma: A previously undescribed and potentially confusing variant. *J Cutan Pathol* (2018) 45:99–103. doi: 10.1111/cup.13068

109. Ardakani NM, Pearce R, Wood BA. Pleomorphic dermal sarcoma with osteosarcoma-like and chondrosarcoma-like elements. *Pathology* (2016) 48:86–89. doi: 10.1016/j.pathol.2015.11.021

110. Argani P, Boyraz B, Oliva E, Matoso A, Gross J, Fridman E, et al. GLI1 Gene Alterations in Neoplasms of the Genitourinary and Gynecologic Tract. *Am J Surg Pathol* (2022) 46:677–687. doi: 10.1097/PAS.0000000000001844

111. Doyle LA, Fletcher CDM. Peripheral hemangioblastoma: clinicopathologic characterization in a series of 22 cases. *Am J Surg Pathol* (2014) 38:119–127. doi: 10.1097/PAS.0b013e3182a266c1

112. Lobo J, Canete-Portillo S, Pena MDCR, McKenney JK, Aron M, Massicano F, et al. Molecular Characterization of Juxtaglomerular Cell Tumors: Evidence of Alterations in MAPK-RAS Pathway. *Mod Pathol* (2024) 37:100492. doi: 10.1016/j.modpat.2024.100492

113. Sotelo-Avila C, Beckwith JB, Johnson JE. Ossifying renal tumor of infancy: a clinicopathologic study of nine cases. *Pediatr Pathol Lab Med* (1995) 15:745–762. doi: 10.3109/15513819509027010

114. Heneghan HM, Martin ST, Casey M, Tobbia I, Benani F, Barry KM. A diagnostic dilemma in breast pathology--benign fibroadenoma with multinucleated stromal giant cells. *Diagn Pathol* (2008) 3:33. doi: 10.1186/1746-1596-3-33

115. Dupont WD, Page DL, Parl FF, Vnencak-Jones CL, Plummer WD, Rados MS, et al. Long-term risk of breast cancer in women with fibroadenoma. *N Engl J Med* (1994) 331:10–15. doi: 10.1056/NEJM199407073310103

116. Fernández-Aguilar S, Noël J-C. [Malignant phyllodes tumor of the breast with osteoclast-like giant cells: a case report]. *Ann Pathol* (2007) 27:31–34. doi: 10.1016/s0242-6498(07)88682-2

117. Liu T, Jiang L, Li J, Sun J, Li H, Gao J, et al. A huge malignant phyllodes tumor of the breast with osteoclast-like giant cells: a case report. *Gland Surg* (2021) 10:1508–1514. doi: 10.21037/gs-20-845

118. Tan H, Zhang S, Liu H, Peng W, Li R, Gu Y, et al. Imaging findings in phyllodes tumors of the breast. *Eur J Radiol* (2012) 81:e62-69. doi: 10.1016/j.ejrad.2011.01.085

119. Yilmaz E, Sal S, Lebe B. Differentiation of phyllodes tumors versus fibroadenomas. *Acta Radiol* (2002) 43:34–39. doi: 10.1080/028418502127347619

120. Makhlouf HR, Abdul-Al HM, Wang G, Goodman ZD. Calcifying nested stromal-epithelial tumors of the liver: a clinicopathologic, immunohistochemical, and molecular genetic study of 9 cases with a long-term follow-up. *Am J Surg Pathol* (2009) 33:976–983. doi: 10.1097/PAS.0b013e31819c1ab3

121. Green C, Spagnolo DV, Robbins PD, Fermoyle S, Wong DD. Clear cell sarcoma of the gastrointestinal tract and malignant gastrointestinal neuroectodermal tumour: distinct or related entities? A review. *Pathology* (2018) 50:490–498. doi: 10.1016/j.pathol.2018.05.001

122. Zhu P, Sun K, Lao IW, Yu L, Bai Q, Zhou X, et al. Expanding the Spectrum of NUTM1-Rearranged Sarcoma: A Clinicopathologic and Molecular Genetic Study of 8 Cases. *American Journal of Surgical Pathology* (2024) 48:930–941. doi: 10.1097/PAS.0000000000002254

123. Liu YJ, Wang W, Yeh J, Wu Y, Mantilla JG, Fletcher CDM, et al. Calcified chondroid mesenchymal neoplasms with FN1-receptor tyrosine kinase gene fusions including FGFR2, FGFR1, MERTK, NTRK1, and TEK: a molecular and clinicopathologic analysis. *Mod Pathol* (2021) 34:1373–1383. doi: 10.1038/s41379-021-00786-x

124. Odate T, Satomi K, Kubo T, Matsushita Y, Ueno T, Kurose A, et al. Inflammatory Rhabdomyoblastic Tumor: Clinicopathologic and Molecular Analysis of 13 Cases. *Modern Pathology* (2024) 37:100359. doi: 10.1016/j.modpat.2023.100359

125. Papke DJ, Dickson BC, Sholl L, Fletcher CDM. Pseudoendocrine Sarcoma: Clinicopathologic Analysis of 23 Cases of a Distinctive Soft Tissue Neoplasm With Metastatic Potential, Recurrent CTNNB1 Mutations, and a Predilection for Truncal Locations. *Am J Surg Pathol* (2022) 46:33–43. doi: 10.1097/PAS.0000000000001751

126. Antonescu CR, Dickson BC, Zhang L, Sung Y-S, Fletcher CD. Unclassified low grade spindle cell sarcoma with storiform pattern characterized by recurrent novel EWSR1/FUS-NACC1 fusions. *Mod Pathol* (2021) 34:1541–1546. doi: 10.1038/s41379-021-00805-x

127. Jakša R, Stružinská I, Kendall Bártů M, Trča S, Matěj R, Dundr P. Clear cell stromal tumor of the lung with multinucleated giant cells: a report of a case with YAP1-TFE3 fusion. *Diagn Pathol* (2023) 18:9. doi: 10.1186/s13000-023-01304-0

128. Fritchie KJ, Torres-Mora J, Inwards C, Thway K, Vaiyapuri S, Jackson R, et al. Xanthogranulomatous epithelial tumor: report of 6 cases of a novel, potentially deceptive lesion with a predilection for young women. *Modern Pathology* (2020) 33:1889–1895. doi: 10.1038/s41379-020-0562-8
